# Pneumatocele after lung transplantation

**DOI:** 10.1186/s44215-023-00030-9

**Published:** 2023-04-11

**Authors:** Masashi Furukawa, Ernest G. Chan, Jenalee N. Coster, Pablo G. Sanchez

**Affiliations:** Division of Thoracic Surgery, Department of Cardiothoracic Surgery, University of Pittsburgh Medical Center, Pittsburgh, PA 15213 USA

**Keywords:** Lung transplantation, Pneumatocele, Primary graft dysfunction, Necrotizing pneumonia, Burkholderia cepacia

## Abstract

Pneumatoceles are a known complication of pneumonia or trauma, especially in young children. A 44-year-old male with pulmonary veno-occlusive disease and pulmonary hypertension underwent double lung transplantation with cardiopulmonary support. The patient had experienced severe primary graft dysfunction and bilateral lower lobe pneumonia. Posttransplant bronchoalveolar lavage fluid identified *Staphylococcus aureus* and *Burkholderia cepacia*. We started trimethoprim-sulfamethoxazole, meropenem, and minocycline. We also switched him to a prone position every 12 h for 3 days. The respiratory condition gradually improved with systemic therapy, prone position, oxygenation with ventilator, and venous-venous extracorporeal membrane oxygenation. Pneumatocele developed at the site of pneumonia. Although the pneumatocele was gradually increasing the size, we decided to continue conservative treatment. The pneumatocele spontaneously ruptured, and asymptomatic pneumothorax developed. We performed percutaneous drainage for pneumothorax, and the pneumatocele resolved. After he was discharged from the hospital, that pneumatocele shrank and disappeared. Pneumatocele can occur at the site of pneumonia after lung transplantation. It may be curable with conservative treatment, but the possibility of sudden rupture and pneumothorax should be considered.

Pneumatoceles are thin-walled, air-filled lung cysts, a known complication of pneumonia or trauma, especially in young children. We report the first case of pneumatocele after lung transplantation. A 44-year-old male with pulmonary veno-occlusive disease and pulmonary hypertension underwent double lung transplantation with cardiopulmonary support. Alemtuzumab was used as the induction immunosuppressive therapy. For maintenance immunosuppressive therapy, tacrolimus, prednisone, and mycophenolate mofetil were used. After transplantation, the patient developed an increased need for oxygen, potentially ischemic reperfusion injury. We decided to put him on venous-venous extracorporeal membrane oxygenation (VV-ECMO). The patient had experienced severe primary graft dysfunction and bilateral lower lobe pneumonia (Fig. 1A). Arterial blood gases revealed pH, 7.37; Pao_2_, 77; Pco_2_, 50; and O_2_ saturation, 92% on F_IO2_ 60%, PEEP 10 cm H_2_O RR 16, TV 400 on a ventilator, and the VV-ECMO circuit was flowing 5.4 L/min with F_IO2_ 100% and sweep 5.5 L/min on postoperative day 9. Posttransplant bronchoalveolar lavage fluid identified *Staphylococcus aureus* and *Burkholderia cepacia*. We started trimethoprim-sulfamethoxazole, meropenem, and minocycline. We also switched him to a prone position every 12 h for 3 days. The respiratory condition gradually improved with systemic therapy, prone position, oxygenation with a ventilator, and VV-ECMO. We decannulated VV-ECMO on postoperative day 20. Pneumatocele developed at the site of pneumonia (Fig. 1B). The patient was weaned from the ventilator on postoperative 30. Although the pneumatocele was gradually increasing the size, the patient’s respiratory condition was improving, so we decided to continue conservative treatment. We confirmed that the bronchoalveolar lavage fluid cultures were negative. He was discharged to a rehabilitation hospital 35 days after lung transplantation. During follow-up, the pneumatocele spontaneously ruptured, and an asymptomatic pneumothorax developed (Fig. 1C). We performed percutaneous drainage for the pneumothorax, and the pneumatocele resolved. Pleural fluid culture obtained from percutaneous drainage was negative. After he was discharged from the hospital, that pneumatocele shrank and disappeared (Fig. 2). This study was approved by our institutional review board (STUDY20050181, approved June 15, 2020), and written consent was obtained from the patient for the publication of this report.

**Fig. 1:**
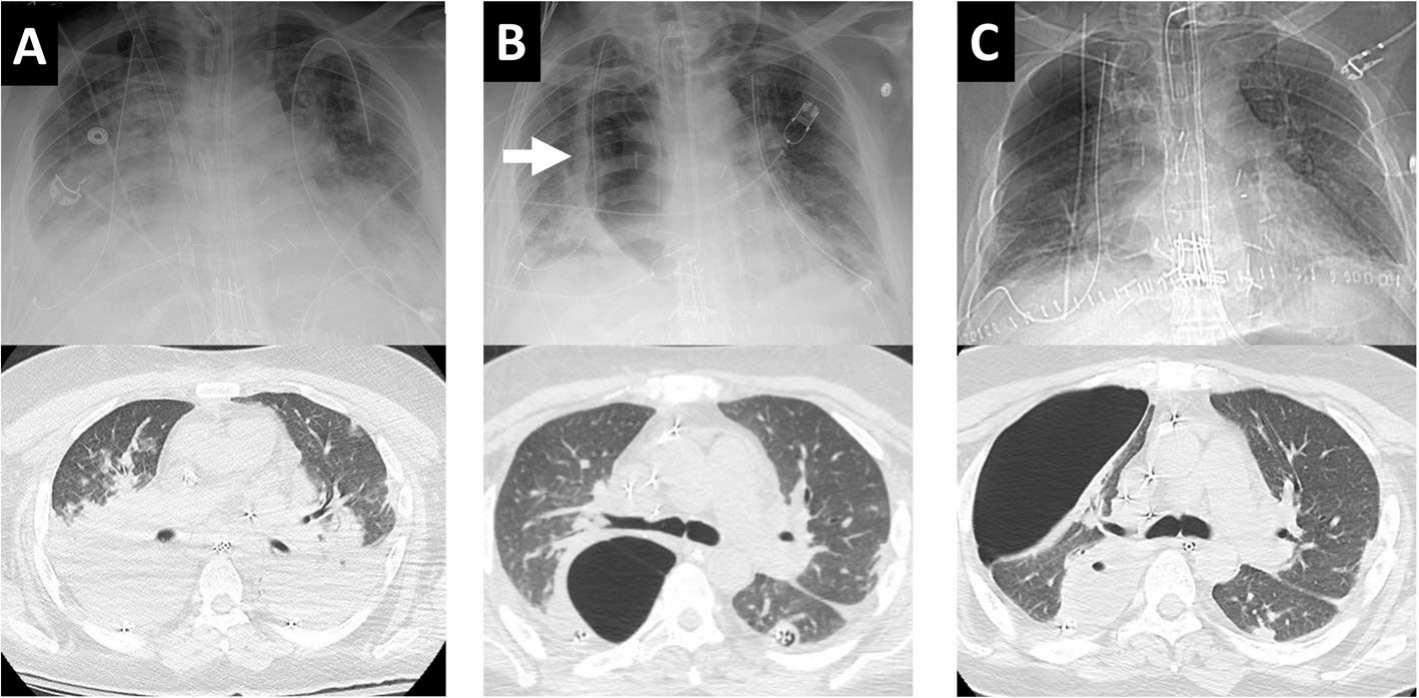
Chest roentgenogram and chest computed tomography. **A** Bilateral lower lobe pneumonia. **B** Pneumatocele. **C** Ruptured pneumatocele and pneumothorax. The arrow showed the pneumatocele

**Fig. 2:**
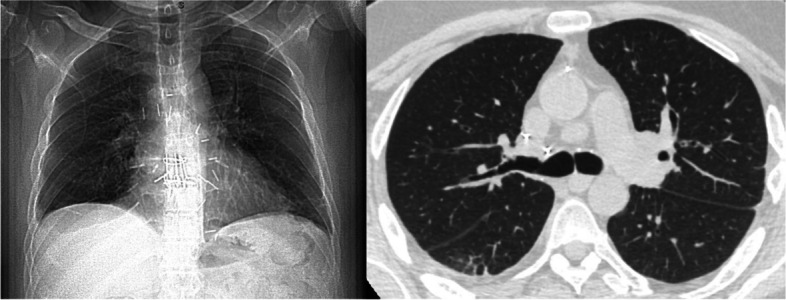
Chest roentgenogram and chest computed tomography after discharge. The pneumatocele has disappeared

## Discussion

Pneumatocele is more common in children and relatively rare in adults. Trauma and inflammation, such as pneumonia, are the most common causes. *Staphylococcus aureus* infections often cause necrotizing pneumonia, which is thought to result in necrosis of the bronchi and check valve mechanism leading to pneumatocele [1]. In addition to *Staphylococcus aureus*, *Burkholderia cepacia* was also detected in this case, which may have caused the fatal cepacia syndrome complicated by necrotizing pneumonia, rapid respiratory decline, and bacteremia [2]. This case had a such severe respiratory failure that oxygenation could not be maintained even with mechanical ventilation and VV-ECMO. Therefore, we believe that *Burkholderia cepacia* significantly impacted this case. *Burkholderia cepacia* is occasionally seen in patients with cystic fibrosis and has a poor prognosis. We modified the antibiotic based on our experience treating patients with cystic fibrosis [3]. The prone position has been reported to be effective in severe respiratory failure and acute respiratory distress syndrome [4]. In the case of bilateral lower lobe pneumonia, as in the present case, the prone position played a significant role in improving respiratory status.

Conservative treatment is the first choice for managing pneumatocele, as most cases resolve spontaneously within 2 months. However, careful follow-up is necessary because persistent infection, hemothorax, severe atelectasis, tension pneumothorax, and development of bronchopleural fistulae may occur [5]. In our case, conservative treatment was chosen, and percutaneous drainage was performed after spontaneous rupture and pneumothorax. Symptoms and imaging findings should be carefully monitored.

## Data Availability

The data are not publicly available due to restrictions on their containing information that could compromise the privacy of the patients.

## Abbreviation

VV-ECMO: Venous-venous extracorporeal membrane oxygenation
